# Total Knee Arthroplasty With Patient-Specific Instrumentation to Correct Severe Valgus Deformity in a Patient With Hereditary Multiple Exostoses

**DOI:** 10.1016/j.artd.2022.04.017

**Published:** 2022-06-24

**Authors:** Urara Sasaki, Masashi Tamaki, Tetsuya Tomita, Seiji Okada

**Affiliations:** a Department of Orthopaedic Surgery, Osaka University Hospital, Osaka, Japan; b Department of Rehabilitation Medicine, North Osaka Housenka Hospital, Osaka, Japan; c Department of Orthopaedic Biomaterial Science, Osaka University, Osaka, Japan; d Grauate School of Health Sciences, Morinomiya University of Medical Sciences

**Keywords:** Total knee arthroplasty, Patient-specific instrumentation, Hereditary multiple exostoses, Valgus deformity, Metal allergy, Constrained prostheses

## Abstract

Patients with hereditary multiple exostosis develop several benign osseocartilaginous bulge lesions throughout the body. A 62-year-old woman presented for evaluation of worsening left knee valgus deformity, and left knee pain. She had been diagnosed with hereditary multiple exostosis at the age of 12 years. Radiographic evaluation of the left knee revealed exostoses that caused continuous bulges from cortical bone at the metaphyseal regions of the femur and tibia as well as extra-articular deformity. We used patient-specific instrumentation to indicate the direction of the stem into curved metaphyseal bone regions and then corrected the patient’s left knee deformity by performing total knee arthroplasty with titanium-constrained prostheses. Soft tissue release was performed with only complete iliotibial band release at a minimum, and stability was obtained.

## Introduction

Hereditary multiple exostosis (HME) is a genetic disease resulting in multiple osteochondromas in which patients develop several benign osseocartilaginous lesions, most often located near the metaphyseal regions of long bones [1]. Such lesions commonly appear as singular entities; only 12% of patients with osteochondroma develop the multiple lesions evident in HME [2]. HME is an autosomal dominant condition caused by a point mutation in the exostosin (EXT) family of genes, which are known as tumor suppressor genes [3]. In the growth plate, EXT1 and EXT2 act on the cartilage membrane to produce parathyroid hormone–related peptide, which contributes to osteoblast differentiation and osteogenesis by interacting with proliferative chondrocytes to control maturation and apoptosis into hypertrophic chondrocytes. When a mutation occurs in EXT1 or EXT2, such that a normal control mechanism is lost, chondrocytes exhibit abnormal proliferation and maturation. Thus, early differentiation and abnormal bone formation occur at the metaphyseal regions of long bones.

The approximate prevalence of HME in White populations (the most widely studied population thus far) ranges from 0.9 to 2.0 individuals per 100,000 [1,4,5]. There is no difference between sexes, and 10% of the affected patients have no family history. The mean age at diagnosis is 3 years; 80% of patients are diagnosed by the age of 10 years [5]. Malignant degeneration to chondrosarcoma reportedly occurs in 0.5% to 25% of patients [6], and EXT1 mutation is a risk factor for severe clinical manifestations [7].

HME causes continuous bony bulges from cortical bone at the metaphyseal regions of long bones and sessile cartilage-capped bony outgrowths throughout the body; these can be diagnosed by clinical and radiographic examinations. When the cartilaginous cap creates an abnormal growth or exhibits excessive uptake in bone scintigraphy of adult patients, malignant degeneration is suspected [8].

The most common site of HME is the knee [6]; such involvement may lead to valgus deformities, restricted range of motion (ROM), and short stature [9]. To our knowledge, no studies have elucidated the cause of exostoses in the knee, but the longitudinal growth rate of long bones is generally higher around the knee than around the proximal femur and distal end of the tibia or fibula [10].

Multiple exostoses, which occur frequently throughout the body in patients with HME, sometimes cause deformities. Shapiro et al. [11] reported that severe deformities involve the forearm and leg, regions in which 2 bones are in a close longitudinal relationship. Extremities consisting of 2 bones exhibit disparate growth rates between longer and shorter bones; thus, a tethering effect of the interosseous membrane and ligaments combined with a disparate growth rate may be causative factors for multiple exostoses, leading to angulation of the longer bone toward the shorter one. Moreover, when 2 exostoses grow on neighboring bones and then fuse together, especially between the tibia and fibula, bone growth becomes restricted [12].

Little is known concerning the surgical management of patients with HME who exhibit severe valgus deformities of the knee. Surgical management of HME affecting the knee encompasses exostoses resection and joint deformity correction by open wedge osteotomy, high tibial osteotomy, and hemiepiphysiodesis [6].

To our knowledge, only 9 patients with knee deformity caused by HME have undergone total knee arthroplasty (TKA); all of these patients had valgus deformity [[13], [14], [15], [16], [17]]. Here, we describe a patient with HME who exhibited severe valgus deformity that was corrected by TKA using patient-specific instrumentation (PSI).

## Case history

### Preoperative evaluation

**Figure 1:**
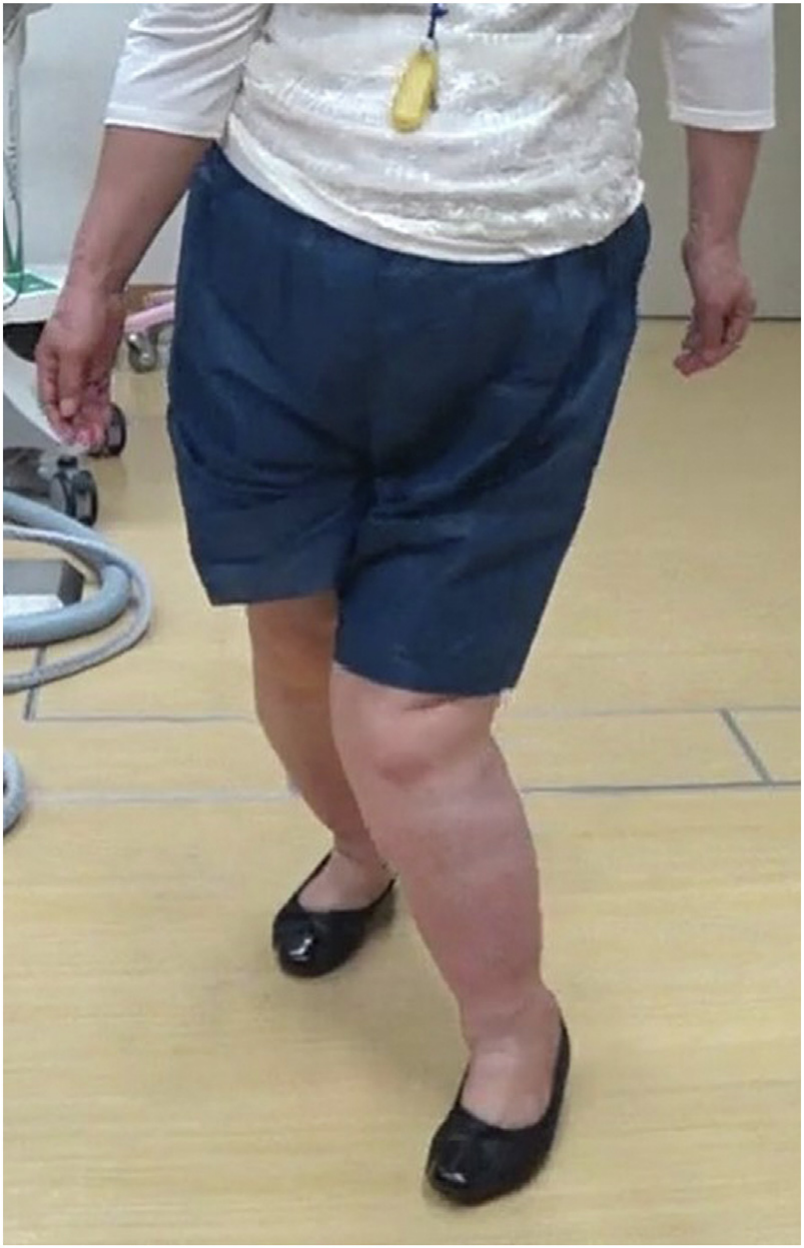
Preoperative standing clinical image. The patient noted a valgus deformity in her left knee that restricted her walking distance to 50 m.

The patient provided informed consent to publish her case in the orthopaedic literature. A 62-year-old woman (weight, 71 kg; height, 148 cm; body mass index, 32 kg/m^2^) presented for evaluation of worsening left knee valgus deformity. She had a known history of HME, metal allergy (palladium, cobalt, and nickel), depression, urinary urge incontinence, and genital herpes. Metal allergy had been diagnosed by patch testing 5 years before she decided to take the operation. She had been diagnosed with HME at the age of 12 years, although she had no corresponding family history. She had undergone multiple surgeries in both legs around the knee for excision of exostoses, beginning at the age of 12 years and continuing until the age of 16 years. At the age of 54 years, she had consulted a doctor because of left knee pain. She received oral medication and therapeutic injections in her knee for 8 years; nevertheless, the pain remained in her left knee. At admission, the patient noted a valgus deformity in her left knee that restricted her walking distance to 50 m; she also had difficulty ascending and descending stairs without a handrail (Fig. 1).

On physical examination, 5 well-healed surgical scars were evident on the medial and lateral aspects of both legs: one in the medial aspect of the left lower leg, 2 in the medial and lateral aspects of the left thigh, and 2 in the medial aspects of the right thigh.

The patient had no pain and only minor ballottement at rest, but she experienced considerable pain during motion and tenderness in the left medial joint space. She also exhibited mild medial-sided opening with valgus stress. The ROM of the right knee was 15-90 degrees. There were no neurovascular abnormalities. The preoperative Knee Society Scores were 8 (objective knee indicators), 4 (symptoms), 16 (patient satisfaction), 12 (patient expectations), and 39 (functional activities).

**Figure 2:**
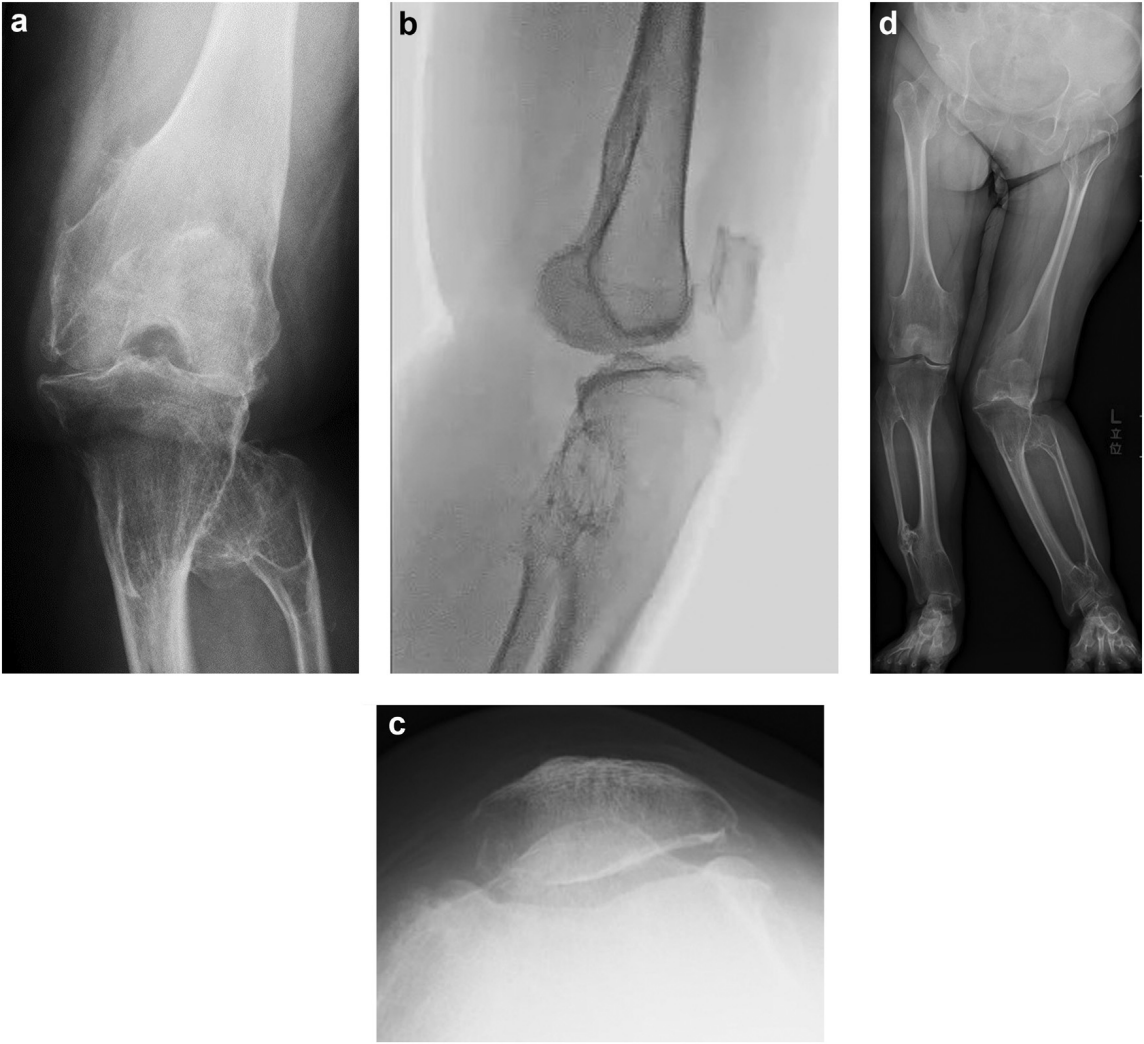
Preoperative (a) anteroposterior, (b) lateral, and (c) skyline views and (d) full-length standing radiographs. (a) The left knee contained exostoses, and end-stage osteoarthritis was present. (b) Femoral posterior subluxation caused by the posterior tibial slope was evident in the lateral view. (c) Patellar dislocation was not present in the skyline view. (d) Full-length views of the lower extremities demonstrated 43-degree valgus deformity of the left knee.

**Figure 3:**
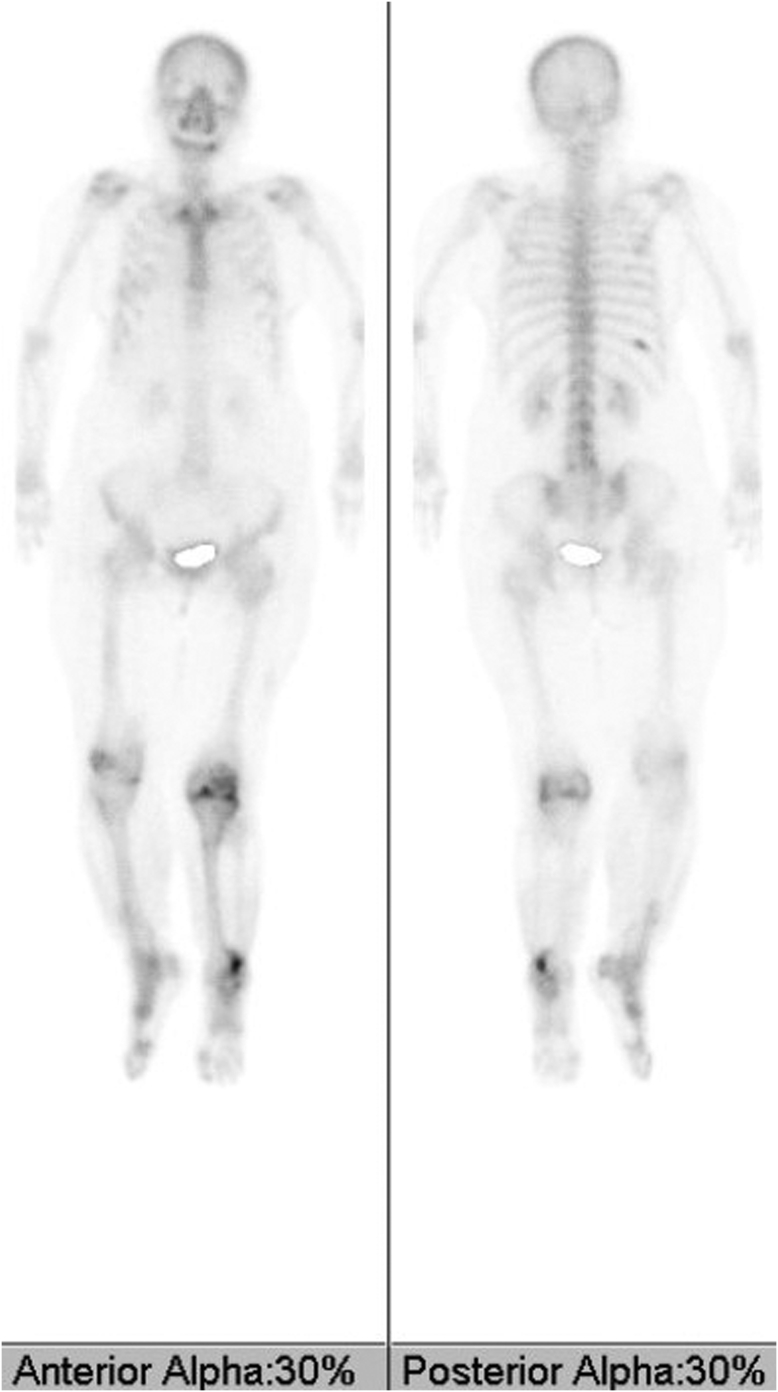
Preoperative bone scintigraphy. Increased uptake was observed in the distal end of the left femur, proximal end of the left tibia, distal lateral end of the left tibia, and right 10th rib.

Radiographic evaluation using 3 views of the left knee revealed exostoses that caused continuous bulges from cortical bone at the metaphyseal regions of the femur and tibia, characteristic of HME. End-stage osteoarthritis was present (most severe in the lateral compartment); this caused severe joint space narrowing and peripheral osteophyte formation. Femoral posterior subluxation caused by the posterior tibial slope was evident in the lateral view. Patellar dislocation was not present in the skyline view. Full-length views of the lower extremities were obtained involving the outwardly curved left tibia and left leg valgus deformity, which was recorded as 43 degrees (Fig. 2a-d). The patient had slight osteoarthritis in both hips and valgus deformity in her left ankle. Magnetic resonance imaging showed anterior and posterior cruciate ligament discontinuity; the medial collateral ligament exhibited a partial elevated signal at the femur adhesion point. Because we suspected other exostoses throughout the body, bone scintigraphy was performed. Increased uptake was observed in the distal end of the left femur, proximal end of the left tibia, distal lateral end of the left tibia, and right 10th rib (Fig. 3).

The identified surgical challenges of this patient were as follows: (1) a severe valgus deformity was present at loading, (2) metal allergies to cobalt and nickel were noted, and (3) an outwardly curved tibia and femoral posterior subluxation were evident. Thus, the following preoperative planning was performed.

(1) Because the patient had a severe valgus deformity at loading and valgus instability, she was presumed to require constrained prostheses.
(2) Because of the metal allergy to cobalt and nickel used in conventional implants, use of titanium prostheses with a constrained hinge design was planned (Kyocera Modular Limb Salvage system [KMLS system]; Kyocera, Kyoto, Japan).
(3) The patient’s outwardly curved tibia suggested the need for TKA with osteotomy. In contrast to conventional implants, KMLS hinge design prostheses have no stem length options (110-mm tibial stem); therefore, we decided not to perform TKA with osteotomy and plate fixation.

To ensure suitable implantation of the tibial component, we decided to place it with a target of 4 degrees of valgus and 7 degrees of posterior slope. We also decided to make a tibial cut in a slight valgus orientation to accommodate the tibial component because of the severe outward curve of the tibia. To counteract this, the distal femoral cut was planned at 5 degrees of valgus alignment. We planned to use a prosthesis with a constrained hinge design to translate the femoral condyle with 33-mm and 30-mm resection of the distal medial and distal lateral femur, respectively.

**Figure 4:**
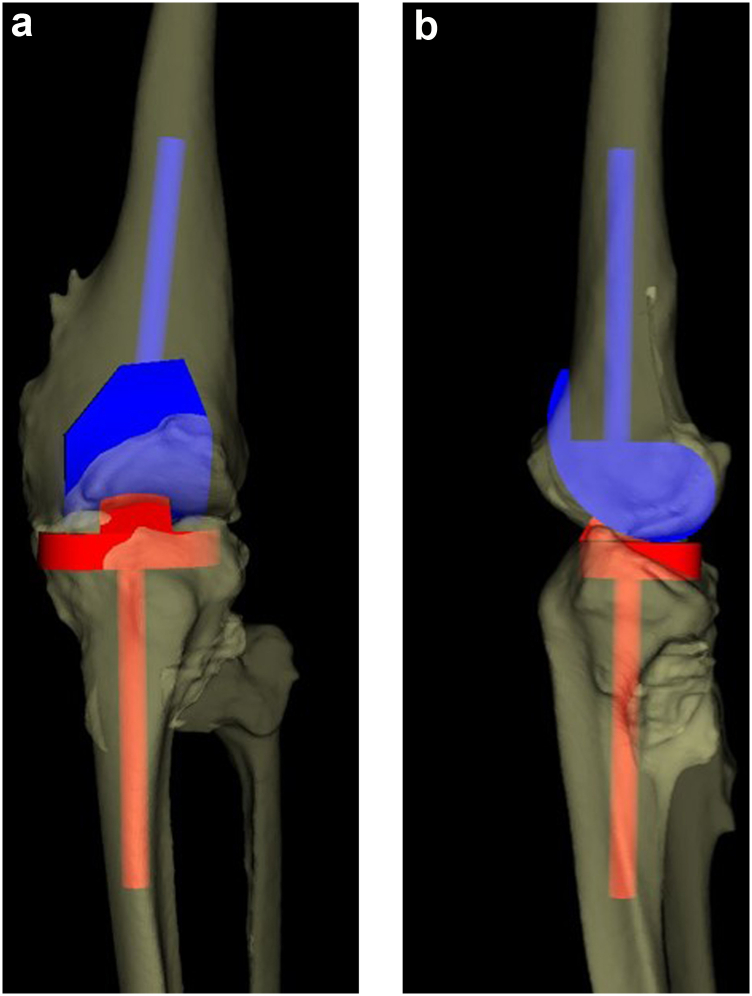
(a) Anteroposterior and (b) lateral computed tomography–based three-dimensional planning of total knee arthroplasty. We planned to use titanium-constrained hinge design prostheses (Kyocera Modular Limb Salvage system [KMLS system]; Kyocera, Kyoto, Japan).

For this purpose, we created PSI for the femur and tibia, indicating the direction of the intramedullary rod; we thus planned TKA using constrained prostheses (Fig. 4a and b).

### Surgical technique

The surgery was performed by the senior authors (M.T., T.T.) with an air tourniquet. An anterior midline incision and medial parapatellar approach were used for exposure. Extensive yellow joint fluid and slight synovitis were observed. Osteoarthritic changes were severe in the medial and lateral femur, but they were slight in the tibial and patellofemoral compartments. Peripheral osteophyte formation was moderate around the femoral, tibial, and patellar compartments. The anterior cruciate ligament was absent, and meniscal remnants were partially split.

**Figure 5:**
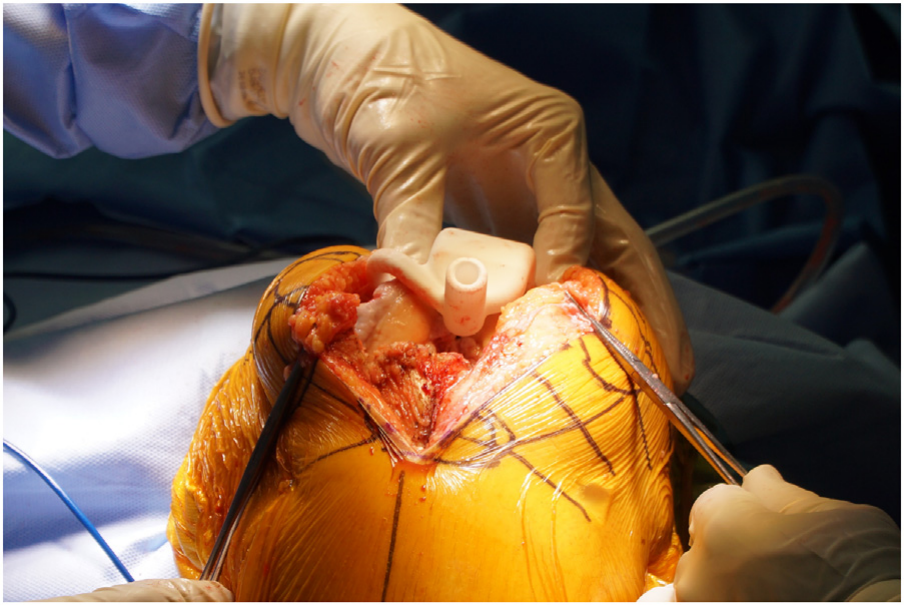
Intraoperative image using patient-specific instrumentation. We created patient-specific instrumentation for the femur and tibia, indicating the direction of the intramedullary rod.

PSI for the distal femur was placed, and a medullary rod was inserted into the femur (Fig. 5). The distal femoral cut was performed as planned. After the meniscal remnants had been excised, attention was directed to the proximal tibia. PSI for the proximal tibia was placed, and a medullary rod was inserted into the tibia. The tibial resection was set at 4 degrees of valgus relative to the mechanical axis of the tibia to avoid over-resection of the medial plateau. Subsequently, the posterior slope was set at 7 degrees with resection depths of 10 mm and 7 mm off the medial and lateral sides, respectively; resection was then performed. We sized the tibial component and performed reaming. We also sized the femoral component, performed the anterior femoral cuts, and performed reaming.

A complete iliotibial band (ITB) release from Gerdy’s tubercle of the femur was necessary to equalize the medial and lateral extension gaps. The flexion and extension gaps were confirmed to be well balanced without medial release.

The patella was resurfaced using measured resection (from 24.0 mm to 13.5 mm). Trial components (size STD for reconstruction of the proximal tibia femoral component with a stem of 124 mm × 10 mm, size SML for the tibial constrained hinge design component, and size STD for the patellar button; Kyocera) were then placed.

The ROM and stability were appropriate with excellent patellar tracking. We placed the components with the accepted construct and then performed cementation and closure. A postoperative neurovascular examination revealed no abnormalities.

After the surgery, the removed tibial exostoses were sent for pathological analysis; the results showed osteochondroma with no indication of malignancy.

### Postoperative evaluation

**Figure 6:**
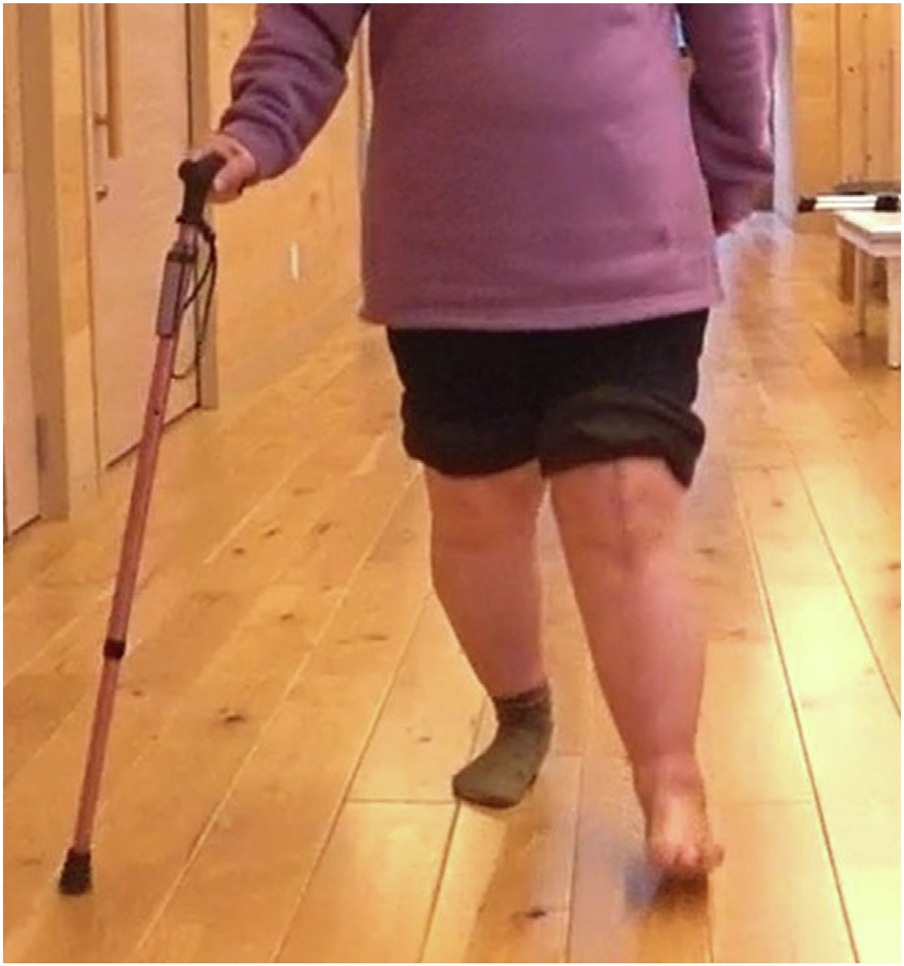
Postoperative standing clinical image. At 6 weeks postoperatively, the patient had no pain in her left knee while ambulating.

**Figure 7:**
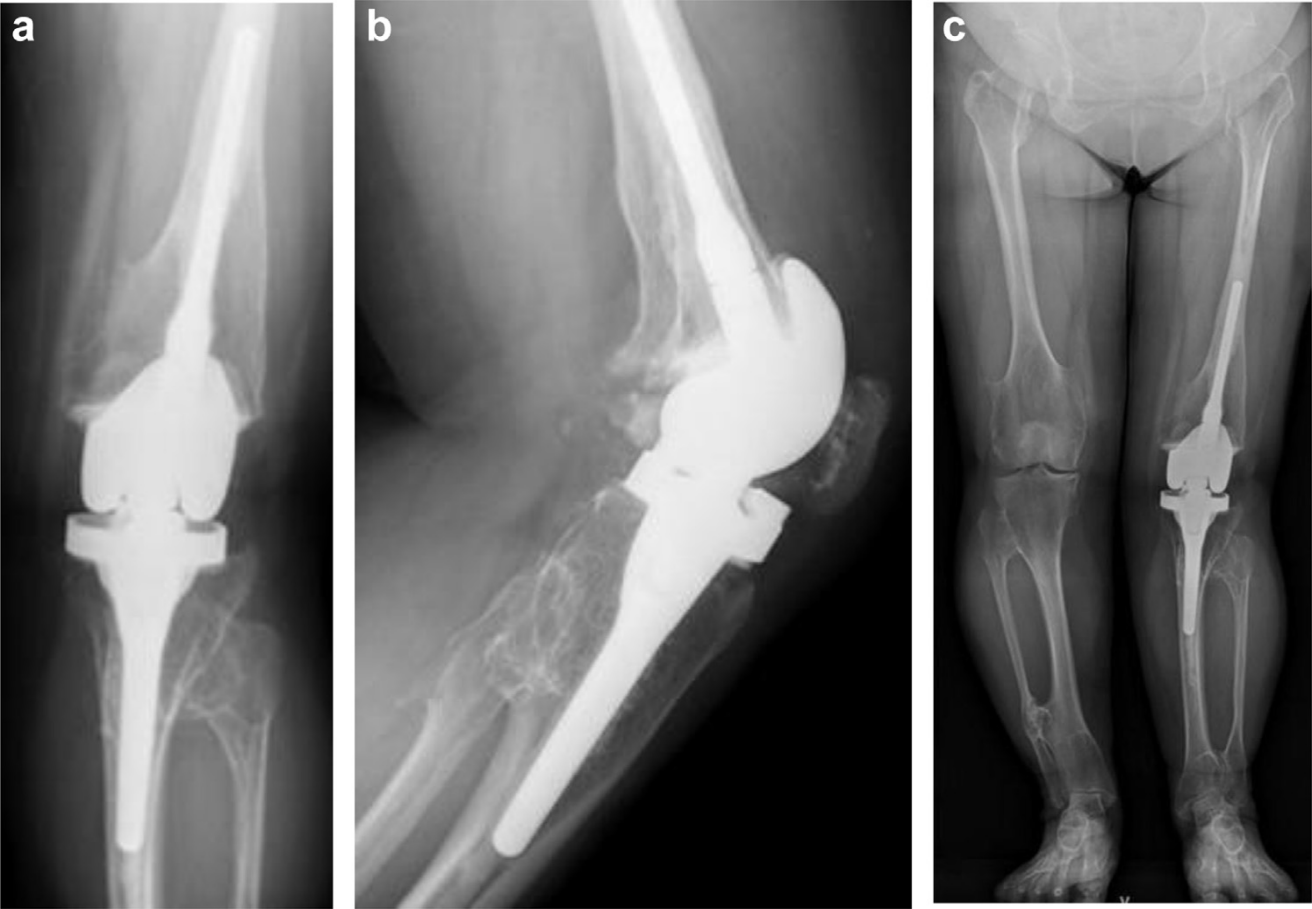
Postoperative (a) anteroposterior, (b) lateral, and (c) full-length radiographs of the left knee at 1-year follow-up. The radiographs demonstrated no evidence of implant loosening, acceptable limb alignment, symmetric pelvic girdles, and 9-degree valgus deformity of the left knee.

The patient had no pain in her left knee while ambulating (Fig. 6), and her walking distance progressed to >2000 m at the latest follow-up (1 year postoperatively). Her ROM was 10-90 degrees without varus or valgus instability. Full-length radiographic views of the lower extremities demonstrated that the mechanical limb alignment was 9 degrees of valgus (this represented a 34-degree correction from the preoperative alignment); additionally, she had symmetric pelvic girdles and no evidence of implant loosening (Fig. 7a-c).

The postoperative Knee Society Scores were 63 (objective knee indicators), 24 (symptoms), 40 (patient satisfaction), 7 (patient expectations), and 57 (functional activities).

## Discussion

From 8% to 28% of patients with HME reportedly have genu valgum because of knee deformities [5,6]. Genu valgum can be caused by an acute increase in exostosis, relative shortening of the fibula, and valgus angulation of the tibia during growth [1]. However, recent studies have demonstrated that the distal femur also contributes to valgus deformity. Clement and Porter [9] reported that increasing numbers of distal femoral exostoses and previous knee surgeries are significantly correlated with valgus deformity. Therefore, the mechanism of valgus deformity caused by general aging is different from the mechanism associated with exostoses during growth.

Detection of valgus deformity at a young age may prevent subsequent progression of valgus by facilitating early orthopaedic osteotomy, such as open wedge osteotomy, high tibial osteotomy, hemiepiphysiodesis [6], and bone extension surgery. Because multiple causes contribute to HME in patients who have become aware of valgus deformity after aging, the deformities become severe and varied; thus, the surgical procedure is increasingly complicated.

Realignment of severe valgus deformity is difficult. For intra-articular changes, performing additional treatments considering the implant selection and soft tissue balance are important. And for extra-articular deformity, it is necessary to consider combining osteotomy [18]. In addition to the valgus deformity, flexion contracture and bone defects of the tibia are often recognized in knees with severe valgum. Therefore, along with normal surgical procedures, there is a need for techniques to release lateral structures (eg, ITB, lateral collateral ligament, popliteus tendon, and posterior cruciate ligament), maintain medial collateral ligament tightness with minimum medial release, and fill the bone defect. In patients with loosened medial structures and persistent instability caused by extensive soft tissue release, the use of constrained prostheses may be useful. With these techniques, surgeons can obtain appropriate alignment and medial-lateral ligament balance.

Surgical management of extra-articular deformity caused by HME in adults has reported techniques including TKA alone [[13], [14], [15]], TKA with osteotomy [16], as well as navigated TKA [17]. To our knowledge, there have been no reports of surgical techniques (eg, TKA using PSI) for management of extra-articular deformity caused by HME.

In patients with severe extra-articular deformities located distally to the joint line, additional extra-articular osteotomy may be required to restore the limb axis and mechanical load. This procedure is expected to improve the knee function and extend prosthesis survival, although it may involve a greater risk of complications [16]. One-stage TKA combined with tibial or femoral osteotomy is potentially riskier with respect to potential complications (eg, instability, nonunion, reoperation, limited ROM, infections, or fracture in the tibial plateau) [[19], [20], [21]]. Reported complications have also included postoperative peroneal nerve palsies, especially in patients with 45-degree valgus deformity [13].

In recent reports, computer-navigated TKA has been shown to improve alignment accuracy [17]. We considered using this navigation method, but we chose to omit it because of the increased risk of pin-site fracture in the distal tibia. Moreover, when using portable navigation systems, only the axial direction can be evaluated; this is not useful for determining the stem direction for severe tibial curvature or the rotation of femoral prostheses. Our patient exhibited an outwardly curved tibia and femoral posterior subluxation; thus, we presumed that PSI was optimal for determining the stem direction and the femoral prosthesis rotation.

The reported benefits of using PSI are its high accuracy and reproducibility, significantly minimal changes in femoral component placement, and shorter operating time [22]. The disadvantages are the lack of significant differences in clinical scores or alignment improvement [22,23] and the increased cost.

In our patient, both intra- and extra-articular factors of severe valgus deformity were present. Therefore, in place of osteotomy, PSI was used to indicate the direction of the stem. Soft tissue release was performed with only complete ITB release at a minimum, and constrained prostheses were used because of severe valgus instability; thus, stability was obtained with slight valgus deformity. Typically, when using a constrained prosthesis, we place the tibial component without a posterior slope. However, in order to ensure suitable implantation of the tibial component, we decided to place it with a target of 4 degrees of valgus and 7 degrees of posterior slope. KMLS hinge design prostheses allowed zero-degree extension, and we therefore intended to avoid anterior impingement despite the posterior slope setting. The hinge type was selected considering the possibility of overextension and release involving all ligaments. Implantation was given priority, and remnant flexion contracture was allowed. In the future, we plan to consider surgical procedures that can achieve realignment, improve ROM, and enhance accuracy.

### Current controversies and future considerations

The ideal management for valgus knee is unclear. The mechanism of valgus deformity caused by general aging is different from valgus deformity associated with exostoses during growth. Because both mechanisms are involved in patients with HME who have become aware of valgus deformity after aging, the deformities become severe and varied; thus, the surgical procedure is complicated.

The use of PSI can aid in the insertion of prostheses in a bowing tibia as well as realignment of severe deformity. To our knowledge, there is limited literature concerning the use of PSI (rather than osteotomy) to indicate the direction of the stem in patients with knee deformity; larger-scale, long-term investigations are necessary.

## Summary

We have described a patient with HME who exhibited severe valgus deformity, which was corrected by TKA using PSI. When treating severe valgus deformity, it is important to consider implant selection, soft tissue balance, and the potential for combined osteotomy with TKA. In our patient, both intra- and extra-articular factors were evident. Rather than osteotomy, PSI in TKA with titanium-constrained prostheses was used to indicate the direction of the stem; soft tissue release was performed with only complete ITB release at a minimum, and stability was obtained.

## Key points

• In patients with HME, the most common site of exostoses is the knee. This may lead to valgus deformities, restricted ROM, and short stature.
• The mechanism of valgus deformity caused by general aging is different from the mechanism of valgus deformity associated with exostoses during growth. Because multiple causes contribute to HME in patients who have become aware of valgus deformity after aging, the deformities become severe and varied; therefore, the surgical procedure becomes complicated.
• Treating valgus deformity is difficult; it is important to perform additional treatments considering implant selection and soft tissue balance with intra-articular changes and to consider combining osteotomy with TKA in patients with extra-articular deformity.
• In our patient, both intra- and extra-articular factors as well as a metal allergy were recognized. Meticulous preoperative planning allowed us to address those problems and achieve stability through TKA using PSI.

## Appendix A Supplementary data

Conflict of Interest Statement for Tomita

Conflict of Interest Statement for Tamaki

Conflict of Interest Statement for Okada

Conflict of Interest Statement for Sasaki
