# Supplementary material for: Total Knee Arthroplasty With Patient-Specific Instrumentation to Correct Severe Valgus Deformity in a Patient With Hereditary Multiple Exostoses
Source: Arthroplast Today. 2022 Jun 24;16:175–81. doi: 10.1016/j.artd.2022.04.017 (PMC9249969; doi:10.1016/j.artd.2022.04.017)
Supplement: Conflict of Interest Statement for Tomita [file mmc1.pdf]

CONFLICT OF INTEREST STATEMENT

American Association of Hip and Knee Surgeons

(Adopted from the American Academy of Orthopaedic Surgeons disclosure statement)

The following form **must be filled out completely and submitted by each author (example, 6 authors, 6 forms). All items require a response. If there is no relevant disclosure for a given item, enter "*None.*"**

Total knee arthroplasty with patient--specific instrumentation to correct a-severe valgus deformity in a patient with hereditary multiple exostoses

1. Royalties from a company or supplier (The following conflicts were disclosed)
   None
   None
2. Speakers bureau/paid presentations for a company or supplier (The following conflicts were disclosed)
   None
   None

3A. Paid employee for a company or supplier (The following conflicts were disclosed)
None
None

3B. Paid consultant for a company or supplier (The following conflicts were disclosed)
None
None

3C. Unpaid consultants for a company or supplier (The following conflicts were disclosed)
None
None

4. Stock or stock options in a company or supplier (The following conflicts were disclosed)
   None
   None
5. Research support from a company or supplier as a Principal Investigator (The following conflicts were disclosed)
   None
   None
6. Other financial or material support from a company or supplier (The following conflicts were disclosed)
   None
   None
7. Royalties, financial or material support from publishers (The following conflicts were disclosed)
   None
   None
8. Medical/Orthopaedic publications editorial/governing board (The following conflicts were disclosed)
   None
   None
9. Board member/committee appointments for a society (The following conflicts were disclosed)
   None
   None

Each author must sign AND print or type his/her name, date and submit a separate form

In addition, one BLINDED Conflict of Interest form (no author names used) should be submitted per manuscript with all author disclosures.

Tetsuya Tomita — [signature] — Aug/9/2021

Author Name (Print or Type) — Author Signature — Date
